# The Photodynamic Properties and the Genotoxicity of Heat-Treated Silicalite-1 Films

**DOI:** 10.3390/ma12040567

**Published:** 2019-02-14

**Authors:** Ivan Jirka, Ivana Kopová, Pavel Kubát, Edyta Tabor, Lucie Bačáková, Milan Bouša, Petr Sajdl

**Affiliations:** 1 J. Heyrovský Institute of Physical Chemistry of the Czech Academy of Sciences, v.v.i, Dolejškova 3, 182 23 Prague 8, Czech Republic; pavel.kubat@jh-inst.cas.cz (P.K.); Edyta.Tabor@jh-inst.cas.cz (E.T.); Milan.Bousa@jh-inst.cas.cz (M.B.); 2 Institute of Physiology of the Czech Academy of Sciences, v.v.i., Vídeňská 1083, 142 20 Prague 4, Czech Republic; ivana.kopova@biomed.cas.cz (I.K.); Lucie.Bacakova@fgu.cas.cz (L.B.); 3 Power Engineering Department, University of Chemistry and Technology, Technická 3, 166 28 Prague 6, Czech Republic; Petr.Sajdl@vscht.cz

**Keywords:** genotoxicity, surface coating, implant material, singlet oxygen

## Abstract

We investigated the use of a supported silicalite-1 film (***SF***) as a promising coating for metallic materials used in the fabrication of prostheses. The role of carbonaceous residua present on high-temperature calcined-***SF*** in generating singlet oxygen for future use as a sterilization method has also been addressed, and the potential genotoxicity of these residua in osteoblast-like cells has been investigated. Calcination of as-synthesized ***SF*** induced the appearance of a rather complicated mixture of aliphatic and aromatic species on its outer surface. A series of variously volatile polycyclic aromatic hydrocarbons (PAH), including naphthalene, fluorene, phenanthrene, anthracene, fluoranthene, and pyrene, were identified in micromole concentrations. Irradiation of these PAHs on calcined-***SF*** immersed in air-saturated chloroform led to the formation of very low concentrations of singlet oxygen. However, an increased level of DNA damage was observed on calcined-SF by immunofluorescence staining of phosphorylated histone H2AX analyzed by flow cytometry.

## 1. Introduction

Appropriate selection of implant biomaterials is a key factor for the long-term success of implants. Implant biomaterials should be selected to reduce the negative biological response, while maintaining adequate function. The relatively long and complicated healing process after the introduction of a joint prosthesis into an organism (i.e., long-term implant osseointegration), and the limited mean lifetime of a joint prosthesis are important problems [1]. The application of prosthetic materials is further complicated by formation of a biofilm with resistant bacteria on its surfaces. These resistant bacteria are difficult to remove by sterilization [2]. In addition, the most widely-used metallic materials, such as stainless steel and titanium-based alloys, mainly Ti6Al4V, are associated with a risk of the release of cytotoxic and allergenic ions [3,4].

One way to improve the biocompatibility of an implant material is to modify its surface in an appropriate manner, e.g., by grinding; by polishing [4,5]; with the use of various oxidative treatments, such as acid and alkaline etching [6,7]; anodic and thermal oxidation [6,8,9]; and coating with a bioactive, chemically stable, and mechanically resistant film [1]. Mordenite Framework Inverted (MFI) zeolite films are candidates for use as a film for this purpose [10,11,12]. 

Supported MFI zeolite films are potentially applicable as a noncytotoxic, robust siliceous porous coating, which can improve the mechanical and anticorrosive properties of the coated material [3,10,11,12,13]. Some researchers have reported good biocompatibility and improved osseointegration of zeolite-coated materials. When applied on Ti6Al4V alloy samples, these films increased the adhesion, proliferation, and osteogenic differentiation of bone-derived and stem cells in vitro in comparison with the bare alloy [11,12,13]. Similarly in vivo, these films significantly enhanced osseointegration and bone regeneration after the implantation of Ti6Al4V samples into rabbit femoral condylar defects [14]. In addition, MFI films showed a greater loading capacity for antibiotics and higher bactericidal activity than conventionally used hydroxyapatite coatings [15].

Medium-pore MFI zeolite with channels defined by ten-member rings belongs to the pentasil zeolite group, and can be prepared in a pure siliceous form (Si/Al → ∞) known as silicalite-1 [16]. Silicalite-1 crystals (***SC****s*) can be grown hydrothermally on the surface of suitable supports in the form of continuous films composed of intergrown zeolite crystals typically varying in thickness from ~10^2^ to ~10^3^ nm. Several procedures have been published for growing these films with various thicknesses and with various orientations of the intergrown crystals relative to the support surface [17]. The most practical procedure is the one-step in situ crystallization method, which can coat surfaces with a complex geometry [17,18,19,20]. The silicalite-1 film (***SF***) is promising as an anticorrosive robust siliceous coating for prosthetic materials [10,11,12,13,14,15].

Hydrothermal synthesis of MFI zeolite often requires the application of molecules of a structure-directing agent (SDA), which fills its micropores. To eliminate SDA, high-temperature calcination is most frequently used [21]. Calcination of MFI zeolite induces thermal degradation of SDA (typically tetrapropyl ammonium (TPA^+^)), which is assumed to proceed via the Hofmann elimination reaction and subsequent β degradation [22,23]. Some of the SDA degradation products (propylene and lower amines) are burned to low molecular species that desorb from the sample. Other products remain bound to the zeolitic phase and form carbonaceous residues from the evolved propylene. Propylene undergoes various reactions, e.g., oligomerization, addition, cyclization, and hydrogen transfer reactions, in which aromatics are produced, together with aliphatic and olefinic species, in the pores of the zeolite [24]. The quantity, the nature, and the stability of the deposits depend on the mode of SDA thermal degradation and on their interaction with the crystal framework. Complete elimination of carbonaceous residues is difficult to achieve, even at high temperatures and with prolonged treatment time [25].

Residual aromatic species were observed on both outermost surfaces of high temperature-treated MFI zeolite (partially graphitized large two-dimensional polycyclic aromatic hydrocarbons (PAHs)) and in the zeolite channels (linear PAHs) [24,26]. These findings were obtained for MFI zeolites with skeletal aluminum, i.e., with acid Brønsted sites, on which dehydrogenation of carbonaceous residues proceeds. In addition, ill-defined aromatic species were also observed in calcined silicalite-1 crystals [25,27]. However, detailed information on concentration and distribution of these PAHs between porous structure of zeolite and its outer surface is missing.

Irradiation of PAH molecules induces the formation of singlet oxygen, i.e., O_2_(^1^Δ_g_), which is a key species in antibacterial photodynamic therapy [28,29]. Photosensitizers such as PAHs immobilized on nano-/microstructured surfaces may under certain conditions preserve their photophysical properties [30]. Photodynamic activity would be promising as a powerful tool for the elimination of resistant bacteria on the surface of the material, which are difficult to remove by any other method. However, the PAHs may not only act as an O_2_(^1^Δ_g_) sensitizer activated by irradiation [31,32], but they could also be cytotoxic or genotoxic. The last both properties would make calcined-SF inapplicable as a prosthetic material.

The cytotoxicity and the genotoxicity of various PAH molecules are issues that have been widely discussed [33]. The toxicity of PAH molecules depends on their metabolic activation. After entering cells, PAH molecules undergo metabolic activation, catalyzed by cytochrome P450 enzymes or by aldo-reductases or keto-reductases, forming reactive metabolites (e.g., dihydrodiol epoxides or *o*-quinones, respectively) [34,35]. These metabolites can interact directly with cell proteins and DNA. One of these proteins is glutathione, an intracellular antioxidant which is depleted by PAH binding [36,37]. This depletion reduces the cellular antioxidant capacity (i.e., cellular detoxification), increases the oxidative stress and disrupts other physiological processes, including Ca^2+^ homeostasis [38].

Another mechanism of PAH genotoxicity is the direct binding of PAH metabolites to the N3 and N7 positions of guanine and adenine in the DNA molecule, creating depurinating DNA adducts [39,40,41]. DNA adducts are known to inhibit the activity of DNA polymerase, resulting in an accumulation of DNA damage [42]. The loss of modified DNA bases generates apurinic sites which, if unrepaired, can cause permanent mutations or DNA double-strand breaks [43]. If these mutations are situated at critical sites, e.g., oncogenes or tumor suppressor genes, they may lead to cellular transformation and the development of cancer. One of the main tumor suppressor genes is p53, with mutations associated with PAH exposure [44,45]. For example, benzo[a]pyrene has been classified as a Group 1 carcinogen by the International Agency for Research on Cancer (IARC) [46].

In this study, the ***a***,***b***-oriented ***SF*** on stainless steel support were prepared. Part of them was calcined at 500 °C, which induced the creation of PAH species. The photodynamic activity and also the cytotoxicity and genotoxicity of PAHs on ***SF*** were then investigated. They depend on various parameters, including the type, volatility, and the dispersion of PAH, its concentration and distribution between the ***SF*** bulk and the ***SF*** surface. The PAHs created on ***SF*** were thus thoroughly characterized using gas chromatography with mass spectroscopy detection (GC–MS), Fourier transform infrared spectroscopy (FTIR), and X-ray photoelectron spectroscopy (XPS). We focused our attention on identifying PAH species in calcined-SF and on estimating their concentration and distribution between the outer surface and microporous structure of the samples. 

## 2. Materials and Methods

### 2.1. Preparation of Silicalite-1 Films (**SF**s) and Silicalite-1 Crystals (**SC**s)

**Figure 1:**
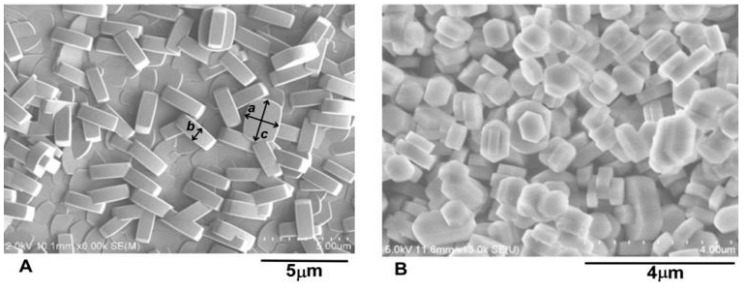
SEM images of ***SF-500*** (**A**) and ***SC-500*** (**B**). The surface of the film is covered by crystals with dimensions ***a*** = 1.0 ± 0.1 μm, ***b*** = 0.5 ± 0.0 μm, and ***c*** = 1.4 ± 0.1 μm (panel A).

Polished stainless steel foil (AISI 316 L, thickness 1 mm), purchased from Goodfellow Metals (GB), was cut into 1 × 1 cm coupons. These coupons were cleaned by sonication in acetone (10 min, 150 W), and were then rinsed in ethanol and in deionized water, and were dried in air. The ***SF****s* were synthesized in situ from the reaction mixture of tetrapropylammonium hydroxide, tetraethylorthosilicalite (both Sigma Aldrich), and deionized water, as described in the literature [19]. The reaction mixture was aged for 2 h. Synthesis proceeded for 3 h in a Teflon-lined autoclave under autogenous pressure at 165 °C. The coupons were oriented upside down during synthesis. This arrangement eliminated sedimentation of the silicalite-1 crystals (***SC****s*) formed during synthesis in the bulk of the reaction slurry. The as-prepared silicalite-1 film (SF-AS) was sonicated in deionized water (10 min, 150 W), and was dried in an air atmosphere at 120 °C. The conditions for this last preparation step were identical with the sterilization of the samples used in the biological experiment. The ***SF****s* consisted of a well intergrown layer of silicalite-1 crystals covered by a discontinuous layer of only partially intergrown ***a***,***b***-oriented coffin-shaped crystals (Figure 1A). We investigated both as-synthesized and heat-treated silicalite-1 films. The heat treatment proceeded as follows: the heating rate was 1 °C min^−1^ up to 500 °C. The sample was maintained at this level for 4 hours, and was then cooled at a rate of 1 °C min^−1^ in a stream of dry air (300 mL min^−1^). No cracks were introduced into the ***SF*** by the heat treatment. The heat-treated sample was designated as SF-500.

The silicalite crystals (***SC****s*) created in the bulk of the reaction slurry during the synthesis of ***SF*** (Figure 1B) were used for Gas Chromatography with Mass Spectrometry detection (GC MS) and Fourier Transform Infrared Spectrosocpy (FTIR) analysis. The external surface of the grained samples was several orders of magnitude larger than the ***SF*** surface, and should contain more carbonaceous residues. The freshly-prepared crystals were extensively washed with deionized water and were dried overnight at 120 °C. The crystals contained 1180 ppm iron (Fe) and 10 ppm aluminum (Al). This sample was designated as ***SC-AS***. An amount of ~0.2 g of this sample was calcined in a quartz boat in a stream of dry air, using the same temperature program as for SF-500. The calcined grained sample was designated as ***SC-500***.

### 2.2. Characterization of **SF**s and **SC**s

#### 2.2.1. Scanning Electron Microscopy (SEM) 

The morphology of the ***SF*** and ***SC*** samples was characterized using an S 4800-I scanning electron microscope (Hitachi, Tokyo, Jpn). Acceleration voltage of 1 kV was applied. 

#### 2.2.2. Gas Chromatography with Mass Spectrometry Detection (GC–MS)

A GC–MS analysis was performed using GC Trace Ultra with a DSQ II mass spectrometer (Thermo Fischer Science, Waltham, MA, USA). To analyze the aliphatic hydrocarbons on the outer surface of the zeolite, the SC-500 (~0.1 g) was suspended in 20 mL of chloroform in a Teflon-lined autoclave at 120 °C for 2 h. A GC–MS analysis on a DB ms 5 m × 0.32 mm column (Agilent, Santa Clara, CA, USA) was carried out with the extract diluted by toluene. The Supelco standard for aliphatic hydrocarbons (Sigma-Aldrich, St. Louis, MO, USA) was utilized for identifying the hydrocarbons in the extract.

The solid SC-500 was analyzed in direct thermal desorption (DTD) mode. The solid was heated in an Optic-2 (Atas, Eindhoven, NL) injection system in a stream of carrier gas (He) to 350 °C (heating rate 15 °C s^−1^). Desorption proceeded directly into GC Trace Ultra. An HPms UI 30 m × 0.25 mm × 0.25 mm film column (Agilent) was utilized. The sample was analyzed before and after evacuation at room temperature (RT) (1 h, ~10^−4^ Pa). 

#### 2.2.3. X-ray Photoelectron Spectroscopy (XPS)

An Omicron Nanotechnology ESCAProbe P spectrometer (Omicron Nanotechnology GmbH, Taunusstein, DE) was used to measure the photoelectron spectra. XPS analysis was performed at a pressure of ~10^−8^ Pa. The X-ray source was monochromatic at 1486.6 eV. The photoelectron spectra were measured at low resolution (survey spectra in the energy region of 0 to 1200 eV with a step size of 0.6 eV) and at high resolution (C 1s, N 1s, and Si 2p spectra in 30 eV scans with a step size of 0.1 eV). The detection angle of the photoelectrons was 90°. The binding energies E_b_ of the C 1s and N 1s photoelectron lines were calibrated by E_b_ of the Si 2p photoelectron line (103.8 eV).

The concentrations of N atoms and variously coordinated C atoms, assigned as ***c(N)*** and ***c(C)***, were evaluated as N/Si and C/Si atomic ratios calculated from the integral intensities of the C 1s, N 1s, and Si 2p photoelectron spectra normalized on the probability of photoemission [47]. The distribution of various carbonaceous species was evaluated from the intensities of the C 1s lines obtained by curve fitting of the C 1s spectrum. A damped nonlinear least-squares fitting procedure was used to distinguish partially resolved lines in the C 1s photoelectron spectra [48]. The fitted lines are marked as ***c(C 1s)^x^***, where ***x*** is the number of lines and is assigned using the NIST database [49].

#### 2.2.4. Fourier Transform Infrared Spectroscopy (FTIR) 

Infrared spectra were recorded using an FTIR Nicolet 6700 spectrometer (Thermo Nicolet Co. Madison, USA) equipped with a liquid N_2_-cooled MCT-B detector. SC-AS and SC-500 were measured in transmission mode, using a heated quartz cell with KBr windows connected to a vacuum. Thin, self-supporting wafers of the samples (8–10 mg cm^−2^) were placed in the holder, which transported the sample from the heating region to the measurement position. FTIR spectra were recorded between 4000 and 400 cm^−1^ at a resolution of 2 cm^−1^ at RT. Hydrated ***SC-500*** was measured first. Then, ***SC-500*** was evacuated under the following conditions (i) 1 h at RT, (ii) 3 h at 250 °C, (iii), overnight at 420 °C, and (iv) overnight at 550 °C. The spectra were normalized relative to the unit height of the most intense skeletal vibration.

The FTIR spectra of ***SF-AS*** and ***SF-500*** were measured using another FTIR Nicolet 6700 spectrometer in the same region and resolution under the ambient atmosphere in reflection mode. For a comparison of samples ***SC-500*** and ***SF-500***, the latter was pretreated at 250 °C for 3 h in vacuum (10^−6^ Pa) ex situ.

### 2.3. Photogeneration of Singlet Oxygen

Luminescence of O_2_(^1^Δ_g_) at 1270 nm was recorded after excitation of both SF-500 and an extract from SC-500 in chloroform by a COMPEX 102 excimer laser (wavelength 308 nm, pulse width ~28 ns) using a Judson Ge diode and interference filters. The signal from the detector was collected in a 600 MHz oscilloscope (Agilent Infiniium, Colorado Springs, USA) and was transferred to a computer for further analysis. The signal-to-noise ratio of the signals was improved by averaging at least 2000 individual traces. The initial part of the traces failed due to the large scattering of the laser light, and it was not used for the evaluation. The resulting kinetics of singlet oxygen luminescence were fitted to a single exponential decay function *I* = *I*_0_ exp(−*t*/τ_Δ_), where τ_Δ_ is the lifetime of O_2_(^1^Δ_g_) and *I* and *I*_0_ denote the luminescence intensity of O_2_(^1^Δ_g_) at time *t* and after excitation (*t* = 0), respectively. Further details can be found in our previous paper [50].

### 2.4. Cell Culture Conditions and an Evaluation of the DNA Damage Response

Samples of stainless steel and stainless steel covered by ***SF-AS*** and by ***SF-500*** were sterilized in a hot-air sterilizer at 120 °C for 2 h, and were inserted into polystyrene 24-well culture plates (diameter 15.4 mm; TPP, Trasadingen, Switzerland). The samples were immediately used for DNA damage analysis, or were evacuated before the evaluation in a vacuum drying chamber (Binder, Tuttlingen, Germany).

The osteosarcoma cell line U-2 OS (ATCC-LGC, Manassas, VA, USA) with wild-type p53 and Rb genes was used to investigate the potential DNA damage to the cells. All samples were seeded with U-2 OS at initial densities ranging from 4200 cells cm^−2^ to 16,100 cells cm^−2^ and were cultured for 7 or 3 days, respectively, in Dulbecco’s Modified Eagle’s Medium (Sigma-Aldrich, St. Louis, MO, USA) supplemented with 10% fetal bovine serum (FBS; Sebak GmbH, Aidenbach, Germany) and gentamicin (40 μgM; LEK, Ljubljana, Slovenia) at 37 °C in a humidified air atmosphere containing 5% CO_2_.

The DNA damage response of the U-2 OS cells was evaluated by immunofluorescence staining analyzed by flow cytometry. Gamma-H2AX antibody (specific to phosphorylated histone H2AX) was used as a marker of DNA double-strand breaks. After 3 and 7 days of cultivation, all samples were transferred to new polystyrene 24-well culture plates and were rinsed with phosphate buffered saline (PBS; Sigma-Aldrich, St. Louis, MO, USA). Cells were then detached by a trypsin-EDTA solution (Sigma-Aldrich, St. Louis, MO, USA), fixed with cold 70% ethanol and were stored at −20 °C for up to two weeks. Fixed cells were rinsed in PBS with 5% FBS and were incubated with Alexa Fluor 488 anti-H2AX-Phosphorylated (Ser139) antibody (5 μg 10–6 cells; Bio-Legend, San Diego, CA, USA; clone 2F3). After 1 h of incubation, the cells were rinsed again and were resuspended in PBS. The stained cells were analyzed using an Accuri C6 Flow Cytometer (BD Biosciences, San Jose, CA, USA).

U-2 OS treated with neocarzinostatin (NCS; 700 ng/mL; Sigma-Aldrich, St. Louis, MO, USA) for 1 h were used as a positive control for the DNA damage response. The cells were fixed 2 h after treatment with NCS. Uncoated microscopic glass coverslips 12 mm in diameter (Menzel Glaser, Monheim am Rhein, Germany) and polystyrene culture plates were used as a reference material. Two samples for each experimental group and time interval were evaluated. To confirm the results, the experiments were performed repeatedly.

## 3. Results and Discussion

### 3.1. Characterization of Carbonaceous Residues in **SF**s and **SC**s

#### 3.1.1. Identification of Carbonaceous Species

The chromatograms obtained by DTD-GC–MS analysis of SC-500 before and after evacuation at RT (Figure S1, Supporting Information—SI) show several PAHs. Naphthalene, phenanthrene, anthracene, fluorene, fluoranthene, and pyrene were detected (Table 1). The concentrations of these PAHs were higher before evacuation due to effective sublimation in a vacuum [51]. Together with the PAHs, a complicated mixture of saturated hydrocarbons was observed by MS in the chloroform extract of sample ***SC-500*** (Figures S2, S3, and SI). According to a comparison of the extract chromatograms with standards, the hydrocarbons can be identified as a series of C_14_–C_32_ alkanes (Figures S3 and SI). However, the retention times in their chromatograms do not exactly match the relevant peaks of the standard. This finding can be explained by the presence of branched alkanes in the mixture.

**Figure 2:**
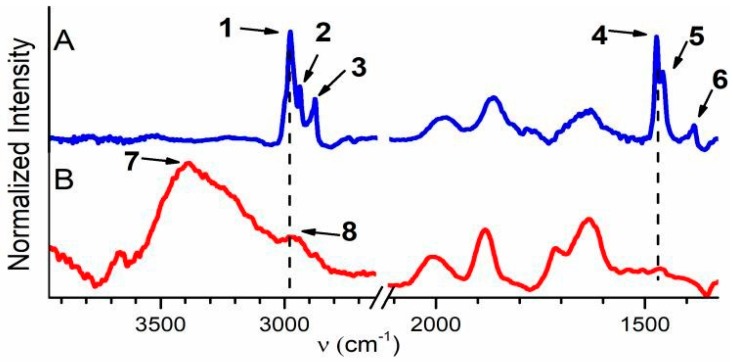
FTIR spectra of sample ***SF-AS*** (**A**) and ***SF-500*** (**B**).

FTIR spectra of samples ***SF-AS***, ***SF-500***, and ***SC-500*** between 4000 and 1500 cm^−1^ are depicted in Figures S4, SI. The regions between ~3900 and 2600 cm^−1^ and ~2100 and 1300 cm^−1^ of these spectra are depicted in Figure 2. Bands **1**–**3** at 2980 cm^−1^, 2943 cm^−1^, and 2881 cm^−1^, respectively, and bands **4**–**6** at 1473 cm^−1^, 1458 cm^−1^, and 1383 cm^−1^, respectively, in the spectrum of SF-AS are attributed to C–H stretching and to C–H asymmetric deformation vibrations of propyl chains of SDA, respectively (Figure 2A) [54,55,56,57,58,59]. In the spectrum of ***SF-500*** (Figure 2B), a strong band—**7**—appeared at ~3394 cm^−1^ (physisorbed water), and bands **1**–**3** were replaced by a weak shoulder **8** at ~2951 cm^−1^, which is characteristic for carbonaceous residues [54,55]. The C–H asymmetric deformation bands of propyl chains of SDA disappeared completely after calcination. Template species were therefore extensively eliminated from ***SF-500***, and only residual carbonaceous species remained [54,55]. 

**Figure 3:**
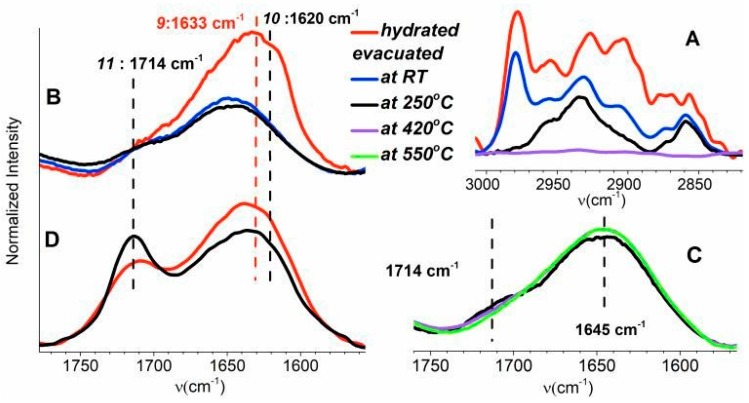
FTIR spectra of the samples with different treatments: C–H stretching vibrations of aliphatic hydrocarbons of ***SC-500*** (panel **A**); the skeletal overtone region of ***SC-500*** (panel **B**) and ***SF-500*** (panel **D**); and expansion of lines **9** and **11** of SC-500 (panel **C**).

The band intensity in a region of C–H stretching vibrations (~3000–2800 cm^−1^) of SC-500 decreases by evacuation at RT and further at 250 °C (Figure 3A). The bands completely disappeared after evacuation at 420 °C in this region. These observations indicate the presence of a mixture of various, mostly saturated, carbonaceous species physisorbed on the outer surface of ***SC-500*** and in its shallow subsurface region.

A comparison of the skeletal vibrations (~2120–1560 cm^−1^) of ***SF-500*** and ***SC-500*** is depicted in Figure S5, SI. Figure 3B,D compare the band of skeletal vibration of these samples in the range between ~1770 and 1550 cm^−1^). Band **9** of ***SC-500*** at 1633 cm^−1^, which overlaps with the band of the skeletal overtone vibration at 1645 cm^−1^, almost disappeared due to evacuation at RT. Further evacuation of sample ***SC-500*** did not substantially affect the intensity of band **9**, and only minor effects were observed. Band **9** was assigned to C atoms coordinated by sp^2^ hybrid bonds [54]. Most of the unsaturated hydrocarbons therefore desorb from sample ***SC-500*** already at RT. A decrease in band **9** was also observed for sample SF-500 (Figure 3D) at 1633 cm^−1^ after evacuation at 250 °C. Band **10** at 1620 cm^−1^ characterized the water bending mode from water adsorbed on silicalite [60]. This band (Figure 3B) is exclusively observed in the spectrum of the hydrated sample. The spectra recorded after evacuation at RT, 250, 420, and 550 °C are free from adsorbed water.

Band **11** at 1714 cm^−1^, observable in samples ***SF-500*** and ***SC-500***, characterized the C=O vibrations originating from an aromatic ring. In the literature, this band is assigned to cyclohexanone [54,55,61]. The higher intensity of band **11**, observed after treatment at 250 °C, indicates the cyclization of hydrocarbon products of template degradation at a higher temperature. Further evacuation of sample SC-500 at 420 °C slightly increased the intensity of band **11** and decreased the intensity of band **10** (Figure 3C). These effects can be explained as a limited aromatization of hydrocarbons in the pores of the zeolite. Complete elimination of the band at 1714 cm^−1^ proceeded at 550 °C (Figure 3C). The results obtained confirm a very low presence of aromatic species in the pores of calcined silicalite-1 crystals.

Most of the carbonaceous residues of ***SF-500*** (***SC-500***) are localized on the outer surface as a mixture of aliphatic and aromatic species. There is only very limited evidence of aromatization of the carbonaceous phase in the voids of silicalite-1, which is readily explainable by the presence of limited Brønsted acid sites in its framework. The limited aromatization of cyclohexanone may be related to the presence of Fe sites in the silicalite-1 framework. We assume that the conversion of aliphatic carbonaceous residues to aromatic compounds proceeds dominantly on the outer surface of ***SF-500***.

#### 3.1.2. Surface Analysis of Carbonaceous Species

**Figure 4:**
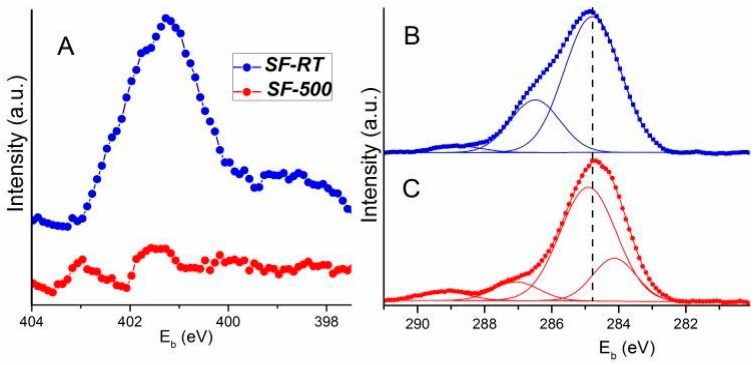
N 1s spectrum of the sample SF-AS (panel **A**). Comparison of the line shape of simulated C 1s spectra of the sample SF-AS (panel **B**) and SF-500 (panel **C**).

XPS experiments were carried out under ultrahigh vacuum (UHV) conditions (10^−8^ Pa), where there is significant desorption of species from the outer surface. As the information depth of XPS in zeolites is ~4–11 nm [62], this method is useful for direct characterization of the shallow subsurface region of ***SF***s. The survey spectra (0–1000 eV) indicate that the samples consist solely of silicon (Si), carbon (C), oxygen (O) (Figures S6 and SI), and the ***SF-AS*** samples also contain nitrogen (N). The N 1s spectrum of sample ***SF-AS*** observed at E_b_ = 402.3 eV (Table 2, Figure 4A) is typical for entrapped TPA^+^ species. The estimated concentration of N in this sample corresponds to the stoichiometric concentration (Table 2). No N 1s spectrum was observed in sample ***SF-500***, i.e., complete elimination of N-containing template species proceeds in this sample by heat treatment (Figure 4A).

The surface region of sample ***SF-AS*** is enriched by a carbonaceous phase. The overall concentration of template carbonaceous species estimated by XPS (C/Si = 0.79) exceeds its stoichiometric value (C/Si = 0.50, Table 2). Only a limited decrease in carbonaceous species was observed by XPS in ***SF-500*** (Table 2). Therefore, a part of the carbonaceous phase remained in the outer surface of ***SF-500***. This finding is in line with our previous results [25]. A non-negligible part of the carbonaceous template species remains in the surface region of silicalite-1 even after prolonged (24 h) calcination [25].

The *C 1s* spectrum was simulated to identify various carbonaceous species in the surface region of the samples. A minimal number of lines with a pseudo-Voight profile were used in the simulation (three lines were used for ***SF-AS*** and four lines for ***SF-500***, Figure 4B,C). The spectrum of ***SF-AS*** was fitted by the *C 1s^2^* line at E_b_ = 284.7 eV with full width at half maximum (FWHM) equal to 2.0 eV, and was fitted by two other high-energy lines (*C 1s^3^* at E_b_ = 286.5 eV (FWHM = 1.8 eV) and very low intensive *C 1s^4^* at E_b_ = 288.8 eV (FWHM = 1.7 eV), Table 2). The *C 1s^2^* line is assigned to C–C (C–H) bonding (i.e., to the C atom coordinated by sp^3^ hybrid bonds). The *C 1s^3^* and *C 1s^4^* lines belong to oxidized C species. The appearance of new low energy *C 1s^1^* in the photoelectron spectrum was induced by calcination. The E_b_ and FWHM values of the remaining lines estimated by this simulation were (within experimental error of ±0.2 eV) identical as in ***SF-AS*** (Figure 4B,C and Table 2). The estimated value of E_b_ (284.1 eV) of the *C 1s^1^* line indicates the presence of C=C coordination [63,64]. The *C 1s* spectrum of SF-500 is dominated by the *C 1s^2^* line of C–C bonding. The XPS analysis thus indicates that sample ***SF-500*** contains predominantly saturated carbonaceous residues (67%). However, there is also a non-negligible unsaturated carbonaceous species (23%). They can be detected in the shallow subsurface region by XPS even under UHV conditions (Table 2). Unsaturated species are summarized in Table 1 according the results: anthracene, fluorene and naphthalene, i.e., species which can accommodate silicalite-1 pores with pore mouth openings ~0.5 nm. The remaining PAH species observed by MS cannot be localized in the pores of silicalite for sterical reasons, and they desorb from the outer ***SF-500*** surface under UHV. The *C 1s^3^* and *C 1s^4^* lines of SF-500 assigned to oxidized C species almost completely disappear after calcination.

Summarizing the results obtained by FTIR, XPS, and GC–MS analyses, we can conclude that most of the SDA is eliminated from ***SF-500*** (***SC-500***) by calcination. The carbonaceous residues remaining in ***SF-500*** are localized on its outer surface and in a shallow subsurface region as a mixture of aliphatic and aromatic species. PAH species (naphthalene, anthracene, pyrene, fluoranthene, and phenanthrene) with various levels of volatility were formed in micromole concentrations. Thermal aromatization is suppressed in the pores of ***SF-500***, and proceeds mainly on the outer surface of SF-500. However, some of the PAHs remained on ***SF*** even after evacuation. 

### 3.2. Singlet Oxygen

Irradiation of PAHs by UV radiation in solution leads to the formation of antibacterial and cytotoxic singlet oxygen O_2_(^1^Δ_g_) with a high quantum yield *Φ*_Δ_ (Table 1) [52]. These results initiated an investigation of the production of O_2_(^1^Δ_g_) by ***SF*** with incorporated carbonaceous residues, using measurements of its characteristic luminescence in the near-infrared region (1270 nm). While chemical probes react with O_2_(^1^Δ_g_), leading to changes in a measurable physical property (absorption, luminescence, or spin signal), this method is not complicated by parasitic chemical reactions [65,66] that have to be evaluated carefully for individual systems. The sensitivity of the luminescence method is limited, due to the small quantum yield of luminescence [67,68].

We tried to detect O_2_(^1^Δ_g_) after pulsed irradiation of ***SF-500*** by UV light in an air and oxygen atmosphere. The pure O_2_(^1^Δ_g_) luminescence was calculated as the difference between the luminescence signal at 1270 nm in an air/oxygen atmosphere and in a vacuum, where O_2_(^1^Δ_g_) cannot be formed. We were not able to separate the luminescence of O_2_(^1^Δ_g_) from the strong background luminescence and scattering due to the low signal-to-noise ratio. Although no O_2_(^1^Δ_g_) luminescence from ***SF-500*** in an air/oxygen atmosphere was directly detected, it can be expected that O_2_(^1^Δ_g_) was formed in a lower concentration with fast decay kinetics, due to the interaction between individual PAH molecules and quenching of the excited states with surface silanol groups of ***SF***. In addition, aggregation and other parasitic photochemical processes when there is a high surface concentration of photosensitizer can significantly reduce the formation of O_2_(^1^Δ_g_) [30,31]. Note that O_2_(^1^Δ_g_) reacts with the PAH photosensitizer itself. Binding of O_2_(^1^Δ_g_) to an aromatic system leads to a loss of conjugation and the formation of endoperoxides [69]. 

**Figure 5:**
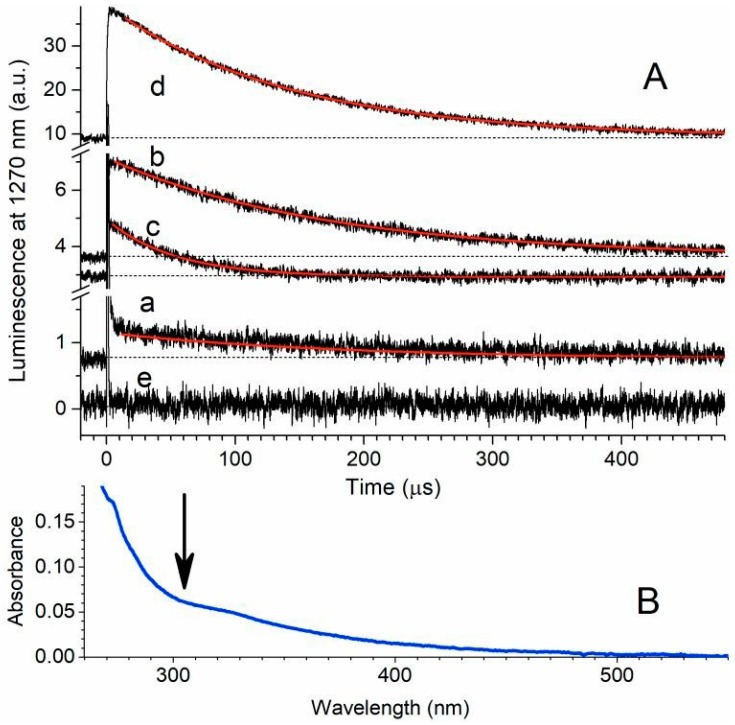
Panel **A**: Kinetics of O_2_(^1^Δ_g_) luminescence after excitation by laser pulse (wavelength 308 nm and pulse width ~28 ns): ***SF*** on steel immersed in air-saturated chloroform (**a**); extract from ***SC-500*** in air-saturated chloroform before (**b**) and after addition of methanol (**c**); standard anthracene in air-saturated chloroform (**d**); and extract from ***SC-500*** in argon-saturated chloroform (**e**, no generation of singlet oxygen) for comparison. Red lines are single exponential fits into experimental data. Individual traces are offset. Panel **B**: UV–Vis spectrum of the extract from ***SC-500*** in chloroform, arrow designates excitation wavelength.

For comparison, the production of O_2_(^1^Δ_g_) by PAH in ***SF***, if any, was more than one order of magnitude lower than for films and materials with previously measured photosensitizers that exhibited strong antibacterial and antibiofouling properties [30,70,71]. In this respect, carbonaceous residues on ***SF*** surfaces do not appear to be suitable for efficient antibacterial treatment and for sterilization of ***SF*** when applied for coating metallic prosthetic materials. We then measured the luminescence of ***SF-500*** immersed in solvents with a long O_2_(^1^Δ_g_), τ_Δ_, lifetime, which have been reported in the literature [52]. We found weak and long-lived luminescence at 1270 nm in air-saturated chloroform, which was assigned to the O_2_(^1^Δ_g_) → O_2_(^3^Σ_g_^−^) transition (Figure 5a). It disappeared after saturation by an inert gas (Figure 5e), and it can easily be separated from shorter background luminescence. The luminescence lifetime (~170 μs) corresponded with τ_Δ_ ~170 μs of photogenerated O_2_(^1^Δ_g_) in chloroform, using standard anthracene as a photosensitizer (Figure 5d), with a high quantum yield of singlet oxygen formation in organic solvents (Φ_Δ_ > 0.5, Table 1) [52].

The amplitude of the O_2_(^1^Δ_g_) luminescence from ***SF-500*** was slightly increased with time after the addition of chloroform. This observation led to experiments with pure extracts of ***SC-500*** with a surface several times bigger than for ***SF***. As expected, the amplitude of O_2_(^1^Δ_g_) luminescence (Figure 5b) was a few times higher than the amplitude of O_2_(^1^Δ_g_) luminescence for zeolite films in chloroform, and it was approximately one order of magnitude lower than the amplitude of O_2_(^1^Δ_g_) luminescence for the standard anthracene photosensitizer (Figure 5d) with the same lifetime of singlet oxygen controlled by the solvent. The addition of methanol accelerated the decay of O_2_(^1^Δ_g_) (Figure 5c), which corresponds with the literature data (τ_Δ_~10 μs for methanol) [52], in comparison with τ_Δ_~170 μs for chloroform. These results clearly show that PAHs were released into the solvents, where they can sensitize the production of O_2_(^1^Δ_g_). The UV–Vis spectrum of the extract of ***SC-500*** with a shoulder in the visible part (Figure 5B) indicates that the formation of O_2_(^1^Δ_g_) can also be photosensitized effectively by daylight. In addition, it shows that carbonaceous species on the surface of ***SC-500*** may consist of PAHs with more than three conjugated aromatic units with an extended window for irradiation up to the blue part of the visible spectrum (Table 1).

### 3.3. The DNA Damage Response

**Figure 6:**
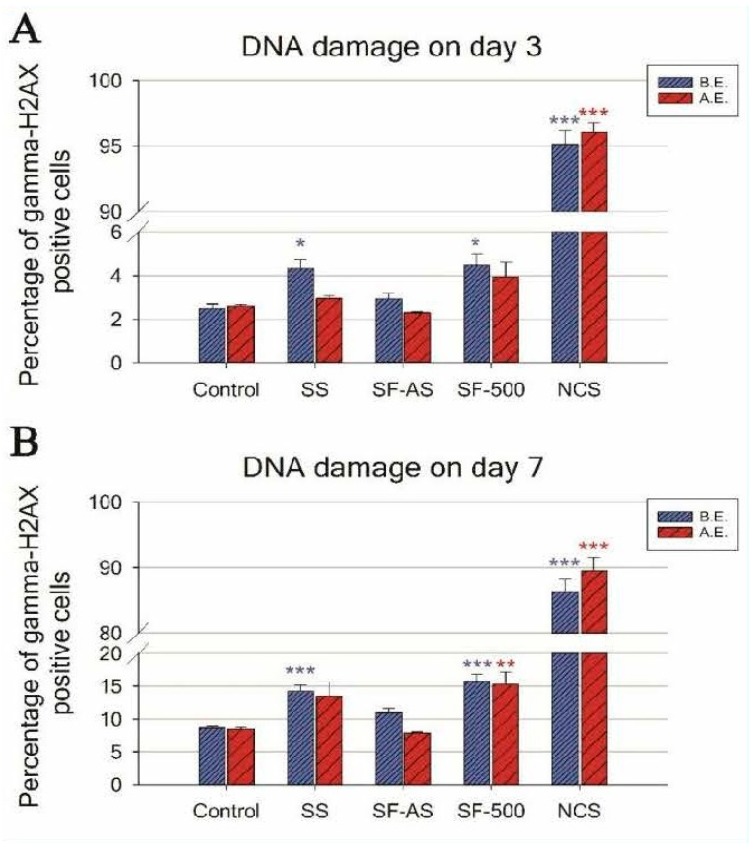
Flow cytometry of the marker of DNA double-strand breaks: gamma-H2AX in human osteoblast-like U-2 OS cells cultured on ***SF-AS*** and ***SF-500*** before and after evacuation. The graphs show the percentage of gamma-H2AX positive cells after 3 (**A**) and 7 (**B**) days of cultivation. SS—stainless steel; NCS—positive control to phosphorylation of histone H2AX (gamma-H2AX) induced by 1 h incubation of U-2 OS in neocarzinostatin (NCS; 700 ng M). The blue columns illustrate the samples before the evacuation (B.E.), while the red columns depict the samples after the evacuation (A.E.). The asterisk represents a significant difference to control with *p* ≤ 0.05 (*), *p* ≤ 0.01 (**), and *p* ≤ 0.001 (***).

The DNA damage response (DDR) was evaluated by flow cytometry, using phosphorylated histone gamma-H2AX, a marker of DNA double-strand breaks. Predictably, during 7-day cultivation of osteoblast-like U-2 OS cells, ***SF-AS*** samples did not cause an increase in DNA damage to cells before or after evacuation (Figure 6).

An evaluation of the DDR in cells cultured on SF-500 with a higher concentration of PAH molecules present on the surface (i.e., before evacuation) revealed increased phosphorylation of histone H2AX. The level of DNA damage was almost 2 times higher than the level in cells grown on the control materials (a polystyrene culture dish or microscopic glass coverslips) for both monitored time intervals—days 3 and 7 (Figure 6, blue columns). 

The evacuation of ***SF-500*** did not fully remove the negative effects on the cells, as residual PAHs remained on the SF-500 surface (Table 1, Figure 4C). Although the increase in DDR on day 3 was not proven to be significant (***SF-500*** did not differ statistically from ***SF-AS*** or from the control SS and GS), the longer 7-day cultivation of cells on evacuated ***SF-500*** promoted significantly higher phosphorylation of histone H2AX (Figure 6B, red columns). These results are in accordance with our previously published study, where ***SF-AS*** samples (as ***SF(RT)***) supported the adhesion and growth of osteoblast-like Saos-2 cells, while cells cultured on evacuated ***SF-500*** had lower cell densities with a poorly developed cytoskeleton [3].

Interestingly, a similar increase in DNA damage was observed in cells cultured on the reference stainless steel without silicalite-1 films (Figure 6, blue columns). Although stainless steel is one of the most widely-used alloys in orthopedic surgery, there is much discussion in the literature about the potential toxicity of this alloy. Several studies have reported an increasing release of metallic ions (e.g., nickel and chromium) from stainless steel over time [72,73,74]. A report comparing several orthopedic alloys showed the highest ion release from stainless steel. Moreover, the viability of human fibroblasts cultured in media with the released metals (obtained from SS submersion for 30 days) dropped rapidly to 4%, while DNA damage increased 7-fold [75]. However, our results did not show increased DDR in cells cultured on SS coated by SF-AS, which means that ***SF-AS*** coating improved the biocompatibility of stainless steel. An increase in DNA damage was found only in silicalite films that undergo calcination (***SF-500***). As has been mentioned, the process of calcination creates PAH molecules on ***SF***.

The cytotoxicity and the genotoxicity of various PAH molecules are often discussed. Since the effect of PAH molecules described in the literature depends on the composition, concentration and exposure time of the PAH, and also on the cell type or organism that is used, contrasting results can be found. The most abundant PAH molecules found on our samples after evacuation were naphthalene, phenanthrene, and anthracene (Table 1).

Older studies performed on macrophages J774A.1 described increased hydroxyl radical production with increased lipid peroxidation, DNA fragmentation, and decreased viability of cells incubated for 24 h at concentrations of naphthalene greater than 200 μM [76,77]. Another report revealed a cell-specific effect of naphthalene on the viability of promyelocytic leukemia HL-60 cells and hepatocellular carcinoma Hep G2 cells. The IC20 values (the inhibitory concentration providing 20% of inhibition) were 150 μM of naphthalene after three hours of exposure in HL-60 cells and 1.7 mM after 48 h of exposure in Hep G2 cells [78]. However, another publication showed much lower IC20 values of naphthalene (approximately 500 μM) for Hep G2 cells after 24 hours of exposure [79]. Moreover, 10 μM of naphthalene was found to induce cell death of human T47D breast cancer cells even after a short exposure time of 1.5 h [80].

Putting these published findings together, we can see that concentrations from a few μM to thousands of μM have been reported to induce cell death in various cell types. However, the lower cell viability caused by an increased rate of cell death is the last stage of the cellular response to irreparable damage, often caused by increased oxidative stress, manifested by the generation of reactive oxygen species (ROS) and resulting in DNA damage to the cells. Therefore, the concentration of naphthalene that is able to induce reparable DNA damage could be much lower. Kapuci et al. [81] reported that a concentration of 10 μM of naphthalene or its metabolites induced increased DNA fragmentation of human lymphocytes without affecting the viability of the cells. Unfortunately, there was no investigation of the lower concentration in this study. The same concentration of other naphthalene metabolites was able to increase ROS generation and the cell death rate in human T47D breast cancer cells after 1.5 h of exposure [80]. Although the concentration of naphthalene detected on our ***SC-500*** after evacuation was much lower (approximately 1.1 μM), the exposure time was much longer (from 3 to 7 days), so an accumulation of DNA damage can be expected over time. Moreover, a recent study confirmed a cell-specific effect of naphthalene, phenanthrene and anthracene, where even very low concentrations (0.31 μM, 0.034 μM and 0.056 μM, respectively) induced a proapoptotic signal (measured by the protein level of Bax and caspase-3) in BeWo placental cells, but not in JEG3 placental cells [82]. This very high concentration range of PAHs that are able to induce DNA damage could explain our result, which indicated a similar level of DNA damage in cells on a calcined film before and after evacuation, i.e., on samples differing in PAH concentration by several times or by an order of magnitude. 

Other PAH molecules found on ***SC-500*** after evacuation were 0.3 μM of phenanthrene and anthracene. These concentrations are almost 10 times higher than the concentrations reported in Drwal’s study mentioned above [82]. Again, different concentrations for various cell types have been reported to induce cytotoxicity. Jacob et al. described the maximum tolerated concentration in the V79 cell line with 30 μM of phenanthrene [79,83]. However, a study evaluating the cytotoxicity of phenanthrene metabolites in placental PEG-3 cells showed that concentrations above 0.1 µM were able to reduce cell viability [80,84]. Cytotoxic and genotoxic effects of phenanthrene and anthracene have been widely investigated in non-mammalian aquatic animals, where concentrations of approximately 0.56 μM cause oxidative stress, lipid peroxidation, nuclear abnormalities, and decreased viability in hemocytes of mussels [81,82,85,86]. A recent publication showed that even a concentration as low as 0.25 nM of phenanthrene was able to induce DNA breaks and lipid peroxidation in erythrocytes of sea bass after 14 days of exposure [87].

In summary, the DNA damage results demonstrate genotoxicity of ***SF-500*** but not of ***SF-AS***. The genotoxicity of high-temperature calcined-SF can therefore be explained by the presence of toxic PAH molecules. However, due to the low formation and the rapid decay of singlet oxygen created by UV irradiation, it is not probable that singlet oxygen participated in the DNA damage observed in our study. In addition, the samples used for DNA damage analysis were not irradiated by UV, and the whole process of cell cultivation was conducted in the dark and without the light exposure that is needed for singlet oxygen generation. In addition, the formation of intracellular ROS, estimated with the use of a DCFDA kit (2′,7′ –dichlorofluorescin diacetate; Abcam, Cambridge, UK), was not higher in the cells on silicalite-1 films containing PAHs than on the control microscopic glass coverslips (data not shown), and therefore cannot be responsible for the observed DNA damage. Therefore, the DNA damage observed in our study could be attributed to direct binding of PAH molecules or their metabolites to DNA and the creation of double-strand breaks in DNA, as is suggested by the presence of phosphorylated histone gamma-H2AX. However, further investigations are needed in order to prove the exact mechanism leading to DNA breaks.

## 4. Conclusions

High temperature (500 °C) calcination of silicalite-1 films liberated its microporous structure, which is potentially prospective in its applicability as an anticorrosive coating of implant metallic material. Simultaneously carbonaceous residues in silicalite-1 films after calcination to 500 °C localized on their outer surface and in a shallow subsurface region as a mixture of aliphatic and aromatic species were detected. Polyaromatic hydrocarbons (naphthalene, anthracene, pyrene, fluoranthene, and phenanthrene) were created in micromole concentrations. Some polyaromatic hydrocarbons (phenanthrene, anthracene, and naphthalene) were entrapped in the micropores of silicalite-1 and thus remained in a shallow subsurface region of the film even after evacuation under high vacuum conditions. Others, including particularly pyrene, fluoranthene, and fluorene, adsorbed on the outer surface of silicalite film, were found to be sufficiently volatile to be released into the environment, where they can sensitize the production of O_2_(^1^Δ_g_) after they have been irradiated by light. However, the estimated concentration of volatile PAHs is too low to be applicable for sterilization purposes. The production of O_2_(^1^Δ_g_) directly on the surfaces, if any, is hindered by interactions between individual PAH molecules, and/or by quenching of O_2_(^1^Δ_g_) by silanol groups. The evaluation of potential DNA damage by silicalite-1 films revealed increased induction of double-strand breaks in osteoblast-like cells cultured on ***SF-500***, but not on ***SF-AS***. The evacuation did not fully diminish the genotoxic effect of ***SF-500*** on cells, due to the presence of residual PAHs species on the surface of the sample. Further investigation of calcined nontoxic anticorrosive ***SF*** coatings of metallic implant materials is required prior their potential use in preclinical trials, consisting in the implantation of ***SF***-coated prosthetic materials into bones of laboratory animals.

## Figures and Tables

**Table 1:** Concentrations of aromatic species found in SC-500 extract and literature values of quantum yields of singlet oxygen formation (*Φ*_Δ_).

Compound	Formula	Concentration (μM)		Φ_Δ_	λ_exc_
		Before Evacuation after Evacuation			
naphtalene	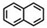	2.3	1.1	-	<290
fluorene	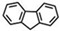	0.6	>0.0	1.00 ^a^	<310
phenathrene	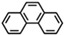	2.7	0.3	0.44 ^a^	<370
anthracene	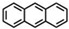	3.5	0.3	0.69 ^b^	<390
fluoranthene	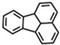	1.6	>0.0	0.50 ^c^	<420
pyrene	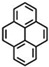	3.8	>0.0	0.79 ^c^	<420

^a^ in hexane [52]; ^b^ in acetonitrile [53]; ^c^ in hexane [52].

**Table 2:** Concentrations of ***c(N)*** and ***c(C)***, binding energies E_b_, and full widths at half maximum (FWHM, in brackets) of photoelectron lines (eV) obtained by simulation of photoelectron spectra.

	*SF-AS*	*SF-500*	Stoichiometry/Assignment
*c(N)*	0.04	0	0.04
*c(C)*	0.79	0.50	0.50
*c(C 1s)^1^*	0	20	
*c(C 1s)^2^*	72	67	
*c(C 1s)^3^*	25	9	
*c(C 1s)^4^*	3	4	
E_b_ (N 1s)	402.3	-	SDA
E_b_ (C 1s)^1^		284.1 (1.5)	Hydrocarbons with sp2 hybridization
E_b_ (C 1s)^2^	284.7 (2.0)	284.9 (2.0)	Hydrocarbons with sp3 hybridization
E_b_ (C 1s)^3^	286.5 (1.8)	287.0 (1.6)	C-O
E_b_ (C 1s)^4^	288.8 (1.7)	289.2 (1.6)	C=O

## Supplementary Materials

The following are available online at http://www.mdpi.com/1996-1944/12/4/567/s1, Figure S1: Chromatograms obtained by DTD-GC-MS analysis of SC-500: naphthalene (A), phenanthrene and anthracene (B), fluorene (C), fluoranthene and pyrene (D). The top in every panel: sample before evacuation, middle: sample evacuated at RT, bottom: standard (Supelco), Figure S2: Mass spectrum of hydrocarbon fractions of the chloroform extract of ***SC-500***, Figure S3: Chromatogram of hydrocarbon fractions of the chloroform extract of sample ***SC-500*** (panel A) compared with the standard mixture of *n*-alkanes (panel B), Figure S4: FTIR spectra of the sample SC-500 (A) and SF-AS, SF-500 (B), Figure S5: Overtone region of infrared absorption spectra of ***SC-500*** (top) and ***SF-500*** (bottom). The spectra of hydrated and evacuated samples at RT and 250 °C are distinguished, Figure S6: Survey photoelectron spectrum of the sample ***SF-500*** composed solely from Si, O, C and in the case of non-calcinated sample also from N.

## Abbreviations

***SF***, silicalite-1 films; SF-AS, ***SF*** as synthesized; SF-500, ***SF*** heated to 500 °C; ***SC***, silicalite-1 crystals; SC-500, ***SC*** heated to 500 °C; PAH, polycyclic aromatic hydrocarbons; SDA, structure directing agent; FWHM, full widths at half maximum; ROS, reactive oxygen species.
