# Supplementary material for: The Photodynamic Properties and the Genotoxicity of Heat-Treated Silicalite-1 Films
Source: Materials (Basel). 2019 Feb 14;12(4):567. doi: 10.3390/ma12040567 (PMC6416588; doi:10.3390/ma12040567)
Supplement: Supplementary file 1 [file materials-12-00567-s001.pdf]

materials

Supplementary Information

The Photodynamic Properties and the Genotoxicity of Heat-Treated Silicalite-1 Films

Ivan Jirka [1,*], Ivana Kopová [2], Pavel Kubát [1], Edyta Tabor [1], Lucie Bačáková [2], Milan Bouša [1] and Petr Sajdl [3]

[1] J. Heyrovský Institute of Physical Chemistry of the Czech Academy of Sciences, v.v.i, Dolejškova 3, Prague 8, 182 23, Czech Republic; pavel.kubat@jh-inst.cas.cz (P.K.); Edyta.Tabor@jh-inst.cas.cz (E.T.); Milan.Bousa@jh-inst.cas.cz (M.B.)

[2] Institute of Physiology of the Czech Academy of Sciences, v.v.i., Vídeňská 1083, Prague 4, 142 20, Czech Republic; ivana.kopova@biomed.cas.cz (I.K.); Lucie.Bacakova@fgu.cas.cz (L.B.)

[3] Power Engineering Department, University of Chemistry and Technology, Technická 3, Prague 6, 166 28, Czech Republic; Petr.Sajdl@vscht.cz

* Correspondence: ivan.jirka@jh-inst.cas.cz

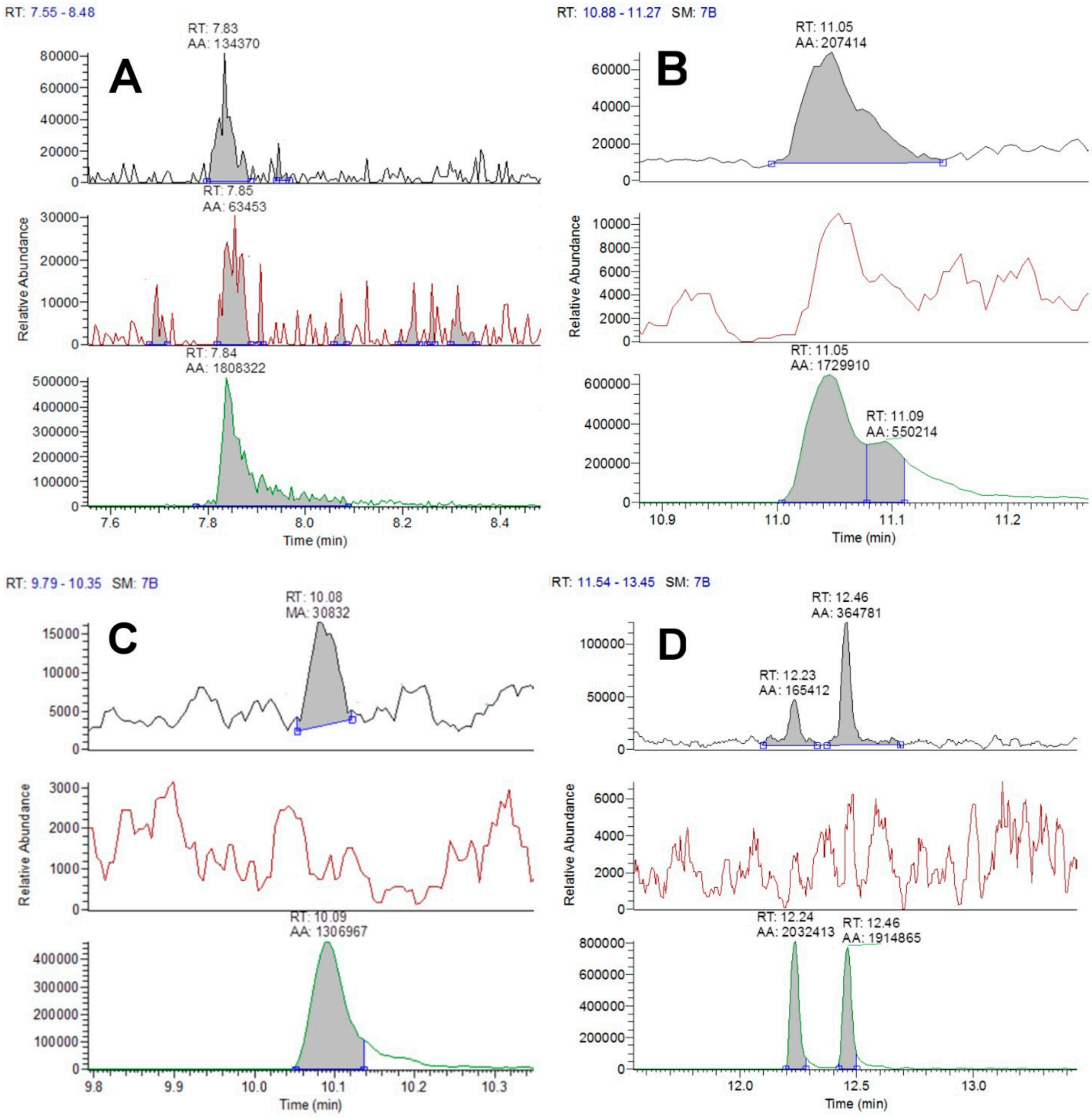

Figure S1. Chromatograms obtained by DTD-GC-MS analysis of ***SC-500***: naphthalene (A), phenanthrene and anthracene (B), fluorene (C), fluoranthene and pyrene (D). The top in every panel: sample before evacuation, middle: sample evacuated at RT, bottom: standard (Supelco).

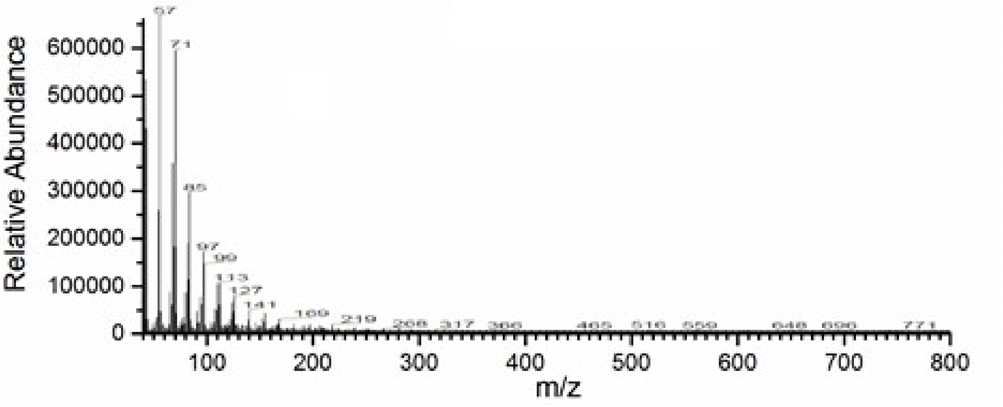

Figure S2. Mass spectrum of hydrocarbon fractions of the chloroform extract of ***SC-500*.**

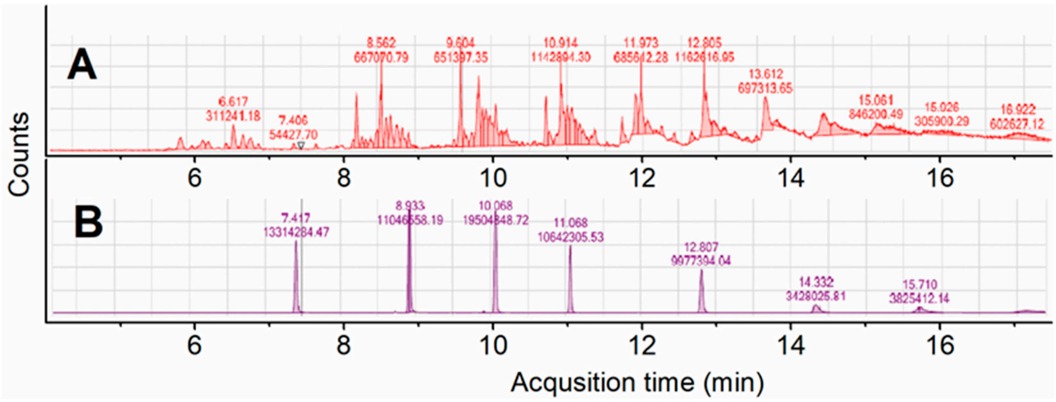

Figure S3. Chromatogram of hydrocarbon fractions of the chloroform extract of sample ***SC-500*** (panel A) compared with the standard mixture of *n*-alkanes (panel B).

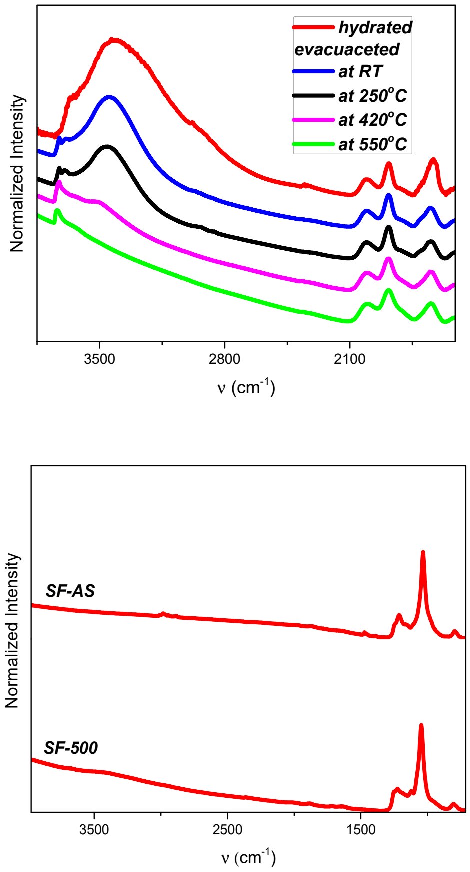

Figure S4. FTIR spectra of the sample ***SC-500*** *(A)* and ***SF-AS***, ***SF-500*** *(B)*.

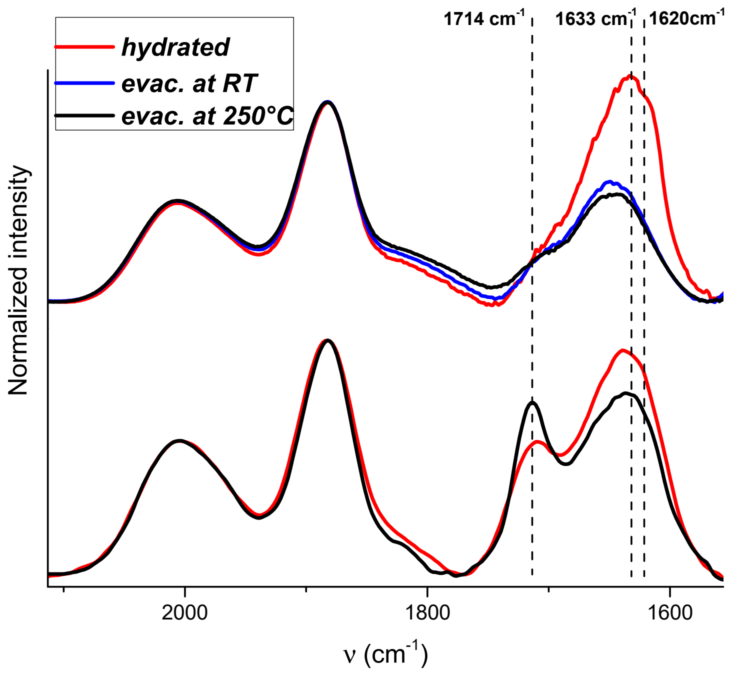

Figure S5. Overtone region of infrared absorption spectra of ***SC-500*** (top) and ***SF-500*** (bottom). The spectra of hydrated and evacuated samples at RT and 250°C are distinguished.

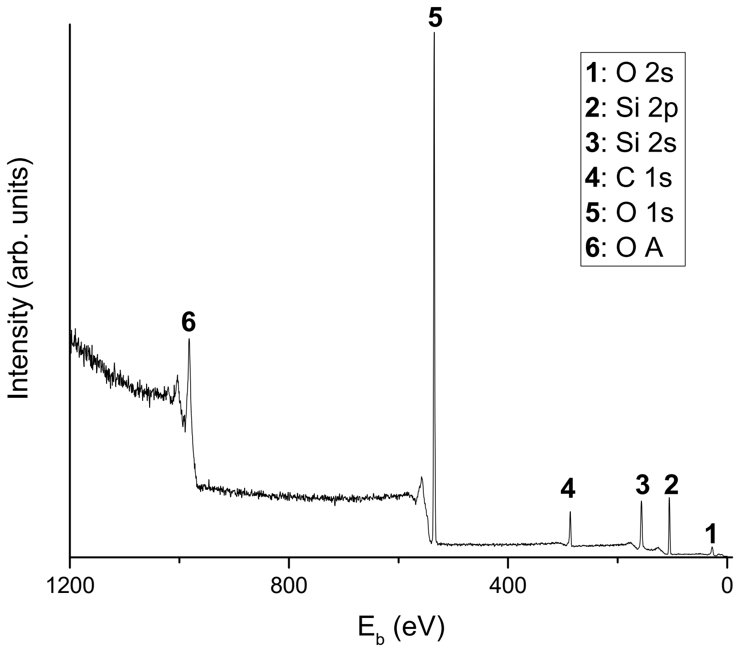

Figure S6. Survey photoelectron spectrum of the sample ***SF-500*** composed solely from Si, O, C and in the case of non-calcinated sample also from N.
